# Financially incentivized knowledge assessments to improve provider compliance with treatment guidelines: a cluster-randomized controlled trial

**DOI:** 10.1186/s13063-022-06129-8

**Published:** 2022-03-21

**Authors:** Günther Fink, György Fritsche, Hadia Samaha, Claude Sese, Gil Shapira

**Affiliations:** 1 Swiss TPH, University of Basel, Basel, Switzerland; 2 World Bank, Washington, DC USA; 3 Ministry of Public Health of DRC, Kinshasa, Republic of the Congo

**Keywords:** Quality of care, Health care worker knowledge, IMCI, Financial incentives, Vignettes, Under-5 mortality

## Abstract

**Background:**

Despite increasing access to health care, under-5 mortality remains high in many parts of Sub-Saharan Africa. Interventions to improve quality of care have mostly focused on additional training for medical staff, but generally shown little impact. We will assess the impact of financially incentivized quarterly provider knowledge assessment on compliance with Integrated Management of Childhood Illness (IMCI) protocols in Congo, DRC.

**Methods:**

Out of a total of 1738 facilities currently receiving results-based financing under an ongoing health financing program, 110 facilities were chosen for this study. All health care workers providing outpatient services to children under age 5 in these facilities will be included in the study. Facilities were randomized with equal probability to control and treatment. Treatment facilities will receive quarterly medical staff knowledge assessments using interactive vignettes. Performance on these vignettes will be rewarded through financial bonus payments to facilities. A baseline survey of health worker knowledge was conducted in 2018. An endline assessment is scheduled to start in the second half of 2021. The primary outcome of interest is health worker compliance with Integrated Management of Childhood Illness (IMCI) guidelines. Compliance will be verified through direct observation of medical staff-patient interactions.

**Discussion:**

This is to our knowledge the first trial assessing whether linking health financing to health care worker performance on knowledge assessments can increase compliance with under-5 case management protocols.

**Trial registration:**

ClinicalTrials.gov NCT04634019. Registered on November 18, 2020.

## Administrative information

Note: the numbers in curly brackets in this protocol refer to SPIRIT checklist item numbers. The order of the items has been modified to group similar items (see http://www.equator-network.org/reporting-guidelines/spirit-2013-statement-defining-standard-protocol-items-for-clinical-trials/).

**Table:** 

Title {1}	Financially incentivized knowledge assessments to improve provider compliance with treatment guidelines: a randomized controlled trial
Trial registration {2a and 2b}.	This trial is registered on *ClinicalTrials.gov* under trial number NCT04634019.
Protocol version {3}	Version 1.4, 22 February 2022
Funding {4}	The study is funded by the Health Results Innovation Trust Fund (HRITF). The interventions are financially supported by HRITF and the International Development Association.
Author details {5a}	Günther Fink, University of Basel and Swiss TPH György Fritsche, World Bank Hadia Samaha, World Bank Claude Sese, Ministry of Public Health of DRC Gil Shapira, World Bank
Name and contact information for the trial sponsor {5b}	Hadia Samaha, World Bank Email: hsamaha@worldbank.org
Role of sponsor {5c}	The funder of this study played no role in the collection of data and will not play any role in the data analysis or interpretation of the results. The final report will be shared with the DRC Ministry of Public Health and the World Bank prior to submission for publication.

## Introduction

### Background and rationale {6a}

Despite major reductions in child health over the past two decades, under-5 mortality remains high, with an estimated 5.6 million deaths globally in 2016 [1]. Major efforts will be necessary to reach the rather ambitious Sustainable Developmental Goal targets for child survival in West and Central Africa, where the under-5 mortality rate was estimated at 98.7 per 1000 live births in 2015 [2].

A growing body of evidence shows that the majority of under-5 deaths occur after children are seen by health care workers [3–5], suggesting major gaps in the quality of care obtained at health facilities [6–8]. Several initiatives have sought to improve the average quality of care through often costly models of training and supervision but generally found little impact on quality of care [9]. Evidence on alternative models such as quality improvement collaboratives or process monitoring remains somewhat mixed [10, 11].

In this trial, we assess the impact of financially incentivized quarterly knowledge assessments on health care worker compliance with the IMCI guidelines in the context of the Democratic Republic of Congo (DRC).

### Objectives {7}

The main objective of this study is to assess whether making health financing streams conditional on health care worker performance on knowledge assessments can increase health care worker compliance with under-5 case management guidelines.

### Trial design {8}

This trial is designed as an open-label parallel two-group cluster randomized trial with a 1:1 allocation ratio.

## Methods: participants, interventions, and outcomes

### Study setting {9}

This trial will be conducted in DRC and is nested within an ongoing results-based financing program in the provinces of Kwango, Kwilu, and Mai-Ndombe in DRC.

Despite the rapid economic growth in recent years and some improvements in some human development indicators, the DRC has some of the worst health and nutrition indicators in the world. Life expectancy was 58 years only in 2016, and under-five mortality was estimated at 88 deaths per 1000 [12]. The maternal mortality ratio is 670 per 100,000 live births.^1^ Chronic malnutrition among children under five is common with an estimated 42.7% stunted according to the 2014 DHS, and almost half of children under five are moderately or severely anemic [13]. Evidence from a recent facility survey suggests that general health care worker compliance with under-5 treatment protocols is very low [14] and that areas with low compliance on average face higher mortality rates [15].

### Eligibility criteria {10}

All health care workers (medical doctors, nurses, and other clinical staff) providing outpatient care to children under age 5 in 110 randomly selected health facilities will be eligible for the study.

### Who will take informed consent? {26a}

Surveyors (study staff) have training either as nurses or medical doctors and will be specifically trained for these assessments. At the beginning of each sick child clinic visit, surveyors will ask both the child’s caregiver and the attending health worker to consent to them sitting in (and documenting) the medical consultation.

### Additional consent provisions for collection and use of participant data and biological specimens {26b}

No additional consents will be collected.

### Interventions

#### Explanation for the choice of comparators {6b}

In order to make the treatment and control areas as comparable as possible, the study focused only on facilities previously enrolled in a results-based financing program. Under this program, quarterly payments are made to all facilities based on the number or pre-specified services delivered as well as a generic quality score. Facilities in the control group thus are identical in terms of the general monitoring and reporting—the only difference is that a part of the quarterly performance-based payments is made conditional on knowledge test performance in the 55 facilities randomly selected for this intervention.

#### Intervention description {11a}

The intervention is a quarterly payment to the health facilities linked to the performance of a randomly selected health care worker on a knowledge-based assessment performed each quarter.

Health facilities in the intervention group will receive a supervision visit each quarter. During the supervision visits, one health care worker is randomly chosen for the knowledge assessments and is tested on two randomly chosen vignette cases. For the intervention, 12 clinical vignettes were created. These vignettes cover typical patient cases such as malaria, diarrhea, and respiratory infections and assess health workers’ ability to correctly diagnose and treat hypothetical questions. All patient scenarios were generic and covered typical symptoms seen in everyday clinical practice without any particular adaptations to local settings.

All medical staff members were informed that there would be a knowledge assessment based on these vignettes every 3 months and that the results of this assessment would determine the total bonus payment made to the facility.

**Fig. 1:**
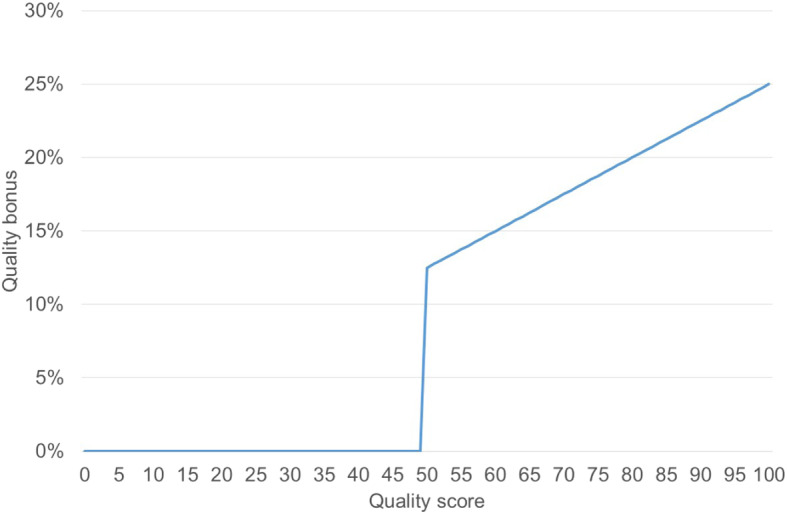
Incentive structure

In order to create a quarterly performance score, the scores on the two vignettes are then averaged. Fifty percent of the overall facility quality score is determined by the general quality checklist that captures the basic structural and process features of the facility. The remaining fifty percent are directly determined by the health care workers’ performance on the knowledge assessments. If the overall quality score is below 50%, no quality payments are made. If the quality score is ≥ 50%, facilities can receive a top-up payment of up to 25% of the quantity-based payments. The total bonus percentage is determined by multiplying the quality score (with ranges between 0 and 100%) with the maximum 25% bonus. As summarized in Fig. 1, any facility with a score below 50% does not get any bonus. A quality score of 50 results in a bonus of 25% × 0.5 = 12.5% − a quality score of 100 results in 25% × 1 = 25% bonus.

**Fig. 2:**
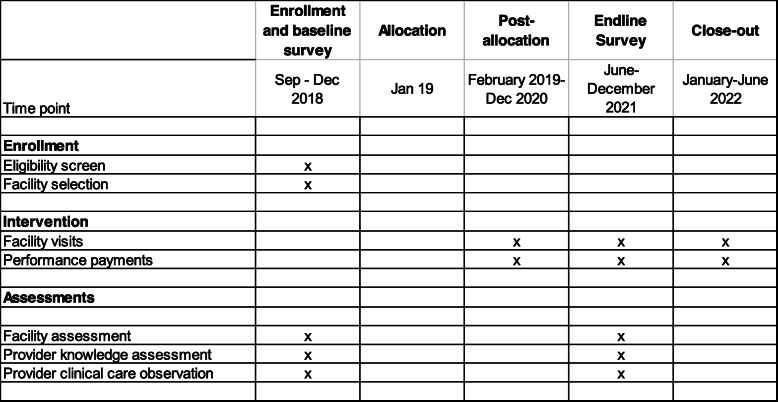
SPIRIT overview over study activities

The knowledge assessments started in the last quarter of 2018 after the completion of the baseline survey. Figure 2 summarizes the overall timeline of the study as well as key measurement points.

#### Criteria for discontinuing or modifying allocated interventions {11b}

No such criteria were established.

#### Strategies to improve adherence to interventions {11c}

The knowledge assessment intervention is embedded in the larger PBF program supervised by the Ministry of Health with support from the World Bank. Completion of the knowledge assessment tests is required in order to determine the quarterly financial transfers to the selected facilities and strictly enforced. A robust monitoring system has been developed for the program as the data is used to determine financial transfers to health facilities. Moreover, the knowledge assessment tests are conducted with tablets, and the data is uploaded to the project portal.

#### Relevant concomitant care permitted or prohibited during the trial {11d}

All subjects participating in the trial will have access to routine health care services throughout the study.

#### Provisions for post-trial care {16}

None.

#### Outcomes {12}

The primary study outcome is compliance with under-5 diagnostic guidelines. Compliance will be measured through up to 8 direct observations of under-5 sick child visits at each facility collected over a 4-day period. For each visit, 10 diagnostic process steps will be measured. Compliance will be defined as at least 8 out of 10 diagnostic processes completed.

Secondary outcomes will be health care worker’s knowledge of IMCI protocols and compliance with the IMCI treatment guidelines. IMCI knowledge will be assessed through a series of vignettes provided to the medical staff. For compliance with treatment guidelines, we will focus on the administration of antibiotics and anti-malarials and referrals to higher level health centers as well as compliance with all diagnostics steps specified in the IMCI guidelines.

Primary and secondary outcomes will be measured as part of a facility survey that will be conducted between May and December 2021. All facilities in the treatment and control areas will be visited by an assessor team for 3–5 days, who directly observe all under-5 sick child visits up to the targeted 8 observations during this facility visit. For smaller facilities, we expect only about one visit per day—for larger facilities, the target of 8 visits can likely be completed within less than two days.

Similar assessments have been conducted through a baseline survey in 2018. These prior assessments will be used to assess baseline balance; the average 2018 quality scores will also be used as controls in adjusted impact estimates.

#### Participant timeline {17}

Baseline facility assessments in the targeted facilities were conducted in 2018. The knowledge assessment was integrated into the quality scoring of selected facilities since the last quarter of 2018. The data collection to determine the intervention’s impact is currently scheduled to be collected in the third and fourth quarters of 2020.

#### Sample size {14}

A total of 110 facilities participate in the trial. Out of these 110, 50% will be randomly selected for the treatment. Within each facility, up to 8 under-5 sick child visits will be observed. We anticipate an average of 6 direct observations in each facility, resulting in a sample of 660 direct observations for our primary outcome. At baseline, compliance with diagnostic protocols was 10%. The study is powered to detect a 15% point increase in diagnostic compliance (from 10 to 25%) with *p* = 0.9, assuming alpha = 0.05 and an intra-class correlation of 0.2 (DEFF of 2).

#### Recruitment {15}

All targeted facilities had previously been enrolled in the larger RBF program and were randomly selected for this sub-study. To collect data on the quality of under-5 consultations, the study team will spend four full days at each facility observing health care workers during their outpatient consultations with children under the age of 5 years. Based on previous studies in this area, we do not anticipate refusals to be a major challenge.

### Assignment of interventions: allocation

#### Sequence generation {16a}

Randomization was done at the health facility (cluster) level. Within each health zone, 50% of health centers were randomly selected for the intervention using a simple random number draw created in the Stata statistical software package by GS.

#### Concealment mechanism {16b}

Given the personal interactions required for the intervention, all treated health care workers will be fully aware of this program. Assessors conducting the direct observations will however not be informed regarding the treatment status of the facilities surveyed.

#### Implementation {16c}

All facilities had previously been enrolled in the PBF program. Random assignment of the intervention was based on a random number draw created by GS. Selected facilities were then visited by the project team and introduced to the intervention.

### Assignment of interventions: blinding

#### Who will be blinded {17a}

Only the outcome assessors will be blinded to the treatment. All health care workers in the intervention group will be fully aware of the program and treatment allocation.

#### Procedure for unblinding if needed {17b}

NA—health care workers will be fully aware of the treatment, and we do not foresee any reasons for unblinding assessors.

### Data collection and management

#### Plans for assessment and collection of outcomes {18a}

The data collection tools are based on tools developed by the Health Results Innovation Trust Fund for the portfolio of impact evaluations of results-based financing projects (https://www.rbfhealth.org/resource/impact-evaluation-toolkit-provides-hands-guidance). The tools that were further calibrated to local settings are part of the baseline survey conducted in 2015. All tools have been tested through pre-study pilots as well as through the baseline survey and the midline survey in 2015. A copy of the direct observation tool is in the Appendix of this document.

#### Plans to promote participant retention and complete follow-up {18b}

We do not foresee any closure of facilities. It is possible that some of the health care workers who were assessed as part of the knowledge tests no longer work at their prior facilities. This will not affect the sample size overall but will be considered when analyzing the data (larger treatment effects expected for medical staff directly selected for the knowledge test).

#### Data management {18}

All data will be collected electronically using the Open Data Kit (ODK) platform. The ODK software allows to enhance data quality through pre-specified range checks as well as internal consistency checks.

Data will be stored at an encrypted local server. Only the PIs will have access to the identified data, and they will de-identify the data before sharing it with the rest of the research team.

#### Confidentiality {27}

All personal information will be removed from data files prior to analysis.

#### Plans for collection, laboratory evaluation, and storage of biological specimens for genetic or molecular analysis in this trial/future use {33}

NA—no biological specimens will be collected or analyzed as part of this trial.

## Statistical methods

### Statistical methods for primary and secondary outcomes {20a}

Multi-level regression models will be used to analyze the primary and secondary study outcomes. The primary outcome (compliance) will be a binary indicator for (observed) the health care worker completing at least 8 out of 10 diagnostic processes foreseen in the IMCI guidelines during an observed consultation. This indicator will be regressed against the randomized facility-level treatment. The empirical model will look as follows:

$$ {Y}_{if}=\alpha +\beta {V}_f+{\varepsilon}_{if} $$

where *Y*_*if*_ is the quality score (1 if compliant, 0 otherwise) of consultation *i* observed in facility *f*, and *V*_*f*_ is an indicator for facilities having received the vignettes intervention (*V* = 1 if yes and 0 otherwise). *β* is the main parameter of interest and captures the changes in the average quality or compliance in response to the intervention.

The secondary outcomes will be continuous knowledge and compliance scores, which will be based on a detailed assessment of medical staff. The unit of analysis for the knowledge outcome is a health care worker rather than a single consultation, with up to 5 health care workers assessed in each facility.

To account for the treatment assignment at the facility level, standard errors will be clustered at the facility level using Huber’s cluster-robust standard errors in all analyses.

Given that facilities were randomly assigned to treatment and control groups, the risk of residual confounding is low—we will however also estimate a series of adjusted models to ensure the robustness of our results as explained in the next paragraph.

### Interim analyses {21b}

No interim analysis is planned.

### Methods for additional analyses (e.g., subgroup analyses) {20b}

To improve the precision of our estimates, we will also estimate the adjusted models where we control for health care worker characteristics (age, training, position, and time at the facility) as well as facility characteristics including facility quality scores at baseline.

In addition, we will conduct a subgroup analysis comparing only medical staff members that were directly assessed in treatment facilities to medical staff in the control group. We will also conduct a stratified analysis at the hospital and health center levels.

### Methods in analysis to handle protocol non-adherence and any statistical methods to handle missing data {20c}

We do not anticipate major missingness on our main outcomes. For the covariates in the adjusted models, we will use multiple imputations by chained equations.

### Plans to give access to the full protocol, participant-level data, and statistical code {31c}

This is the only protocol that will be published for this study. The statistical code will be made available upon publication.

### Oversight and monitoring

#### Composition of the coordinating center and trial steering committee {5d}

There is no formal trial steering committee. The interventions are implemented by a project implementation unit of the Ministry of Public Health with supervision and support of GFritsche the World Bank DRC health team. All aspects related to the data collection and analysis are managed by GS in collaboration with GFink.

#### Composition of the data monitoring committee, its role, and reporting structure {21a}

This project does not have a data monitoring committee.

#### Adverse event reporting and harms {22}

Given that the primary objective of this project is to improve health care worker knowledge and compliance with the protocols, no major harm is anticipated. Health care worker concerns regarding the program can be directly shared with the implementation team.

#### Frequency and plans for auditing trial conduct {23}

There is no trial audit foreseen.

#### Plans for communicating important protocol amendments to relevant parties (e.g., trial participants, ethical committees) {25}

In the unlikely event of a major intervention change, such changes will be directly added to the trial registration and communicated to the local ethics board.

#### Dissemination plans {31a}

The results of this trial will be directly reported to the national ministry of public health and also presented at public conferences. We also hope to publish the final results in a high-impact peer-reviewed journal.

## Discussion

This is to our knowledge the first trial investigating the effectiveness of financially incentivized knowledge vignettes on medical staff behavior. The 12 vignettes used in this study were developed by the study team specifically for this project and were designed to cover the most common infectious diseases, but of course do not cover the full mix of cases faced by the health care workers. It is also not clear if facilities directly reward or punish medical staff performance on these tests—no guidance was given to facilities with regard to this—detailed data on this as well as other aspects will be collected during endline.

## Trial status

A baseline survey was completed in 2018. Random selection of facilities was done in 2018. Enrollment for the outcome assessment is scheduled to start in late 2020 and will last for 5–7 months.

Protocol version 1.4, 22 February 2022

## Appendix

Direct observation tool

**Table:**

***Section 1 : Identification (Posez directement la question à l’agent)***				***NOTER LES RÉPONSES***			
**(1.01)**	Nouveau patient	Oui	1	|__|			
		Non	2				
**(1.02)**	SAISIR LE NUMÉRO DE SUIVI DU PATIENT			|__||__|			
**(1.02a)**	NOM/PRÉNOM DU PATIENT			_____________________			
**(1.03)**	Sexe du patient	Masculin	1	|__|			
		Féminin	2				
**(1.04)**	Numéro de suivi de l’agent de santé (Questionnaire F1)			|__||__||__||__||__||__||__|			
**(1.04a)**	Nom/Prénom de l’agent de santé			_____________________			
**(1.05)**	Sexe de l’agent de santé	Masculin	1	|__|			
		Féminin	2				
**(1.06)**	Type d’ agent	Médecin	1	|__||__|			
		Infirmier(ère) L2	2				
		Infirmier(ère) A2	3				
		infirmier(ère) A1	4				
		technicien de labo	5				
		Sage-Femme / accoucheur	6				
		Autre: Specifiez	7				
***Section 2 : Prise des antécédents et examen***							
**(2.01)**	Heure de début de la consultation ex. 7 h est 0700, 8 h 30 est 0830 et 19 h est 1900. *Veuillez retenir que la consultation peut se dérouler en deux phases.*					|__||__||__||__|	
**(2.02)**	L’agent de santé salue-t-il le patient et/ou l’accompagnateur du malade ?	Oui	1			|__|	
		Non	2				
**(2.03)**	L’agent de santé se lave t-il les mains avec du savon et de l’eau du robinet ou seau avec robinet avant d’examiner le patient (utilisation de desinfectant, **GEL**) ?	Oui	1			|__|	
		Non	2				
**(2.04)**	A t-il demandé l’âge du patient ?	Oui	1			|__|	
		Non	2	►	**(2.06)**		
		pré-rempli	3				
**(2.05)**	Quel est l’âge du patient ? **Notez 99 si l’accompagnateur ne sait pas. Remplissez "00" mois si l’enfant a moins d’un mois, et "00" années si l’enfant a moins d’une année.**	a. Années				|__|__|	
		b. Mois				|__|__|	
**(2.06)**	L’agent de santé a-t-il demandé la nature du mal (motif de la présente consultation)?	Oui	1			|__|	
		Non	2	►	**(2.09)**		
**(2.07)**		Diarrhée	1			|__||__|	
	UN SEUL SYMPTOME/PLAINTE **PRINCIPAL** POSSIBLE	Fièvre (incl. paludisme)	2				
		Toux/difficulté respiratoire	3				
		Dermatose (maladie de la peau)	4				
		Difficulté d’avaler, douleur à la déglutition (Amygdalite / angine)	5				
		Douleur à l’oreille (Otite)	6				
		Blessure	7				
		Convulsion	8				
		Perte de conscience	9				
		Asthenie physique(fatigue)	10				
		Vomissement	11				
		Autre à preciser	97				
**(2.08)**	MARQUEZ **TOUS LES AUTRES** SYMPTOMES MENTIONNES PAR L’ACCOMPAGNATEUR	a.Diarrhée				|__|	
		b.Fièvre				|__|	
		c.Toux/difficulté respiratoire				|__|	
		d.Dermatose (maladie de la peau)				|__|	
		e.Difficulté à dégluttir, douleur à la déglutition (Amygdalite / angine)				|__|	
		f.Douleur à l’oreille (Otite moyenne)				|__|	
		g.Blessure				|__|	
		h.Convulsion				|__|	
		i.Perte de conscience				|__|	
		j.Asthenie physique(fatigue)				|__|	
		k.Vomissement				|__|	
		l.Autre à preciser				|__|	
	NOTER « 1 » POUR OUI ; « 2 » POUR NON POUR CHACUNE DES OPTIONS SUIVANTES	**Oui**	**Non**				
**(2.09)**	L’agent a-t-il demandé la durée de probleme ?	1	2			|__|	
	**Si (2,09)=1, Depuis combien de temps l’enfant est-il/elle malade ? (99 ne sait pas)**	a. jours				|__|__|	
		b. Mois				|__|__|	
**(2.10)**	Un agent de la formation sanitaire a-t-il pesé l’enfant ?	1	2			|__|	
	**Si (2,10)=1, Poids de l’enfant?**	Poids en grammes					
		(99999 ne sait pas)				|__|__||__|__||__|	
**(2.11)**	Quelqu’un de la formation sanitaire a-t-il mesuré la taille de l’enfant ?	1	2			|__|	
	**Si (2,11)=1, Taille de l’enfant?**						
		Taille en cm (999 ne sait pas)				|__|__||__|	
**(2.12)**	Un agent de la formation sanitaire a-t-il pris la température de l’enfant?	1	2			|__|	
	**Si (2,12)=1, Temperature de l’enfant?**	Temperature en degré celsius (99.9 ne sait pas)					
						**|__|__|.|__|**	
**(2.13)**	L’agent de santé a-t-il demandé si l’enfant peut boire ou prendre du lait maternel/biberon ?	1	2			|__|	
	**Si (2,13)=1, L’enfant peut boire ou prendre du lait maternel / biberon?**	Oui	1			|__|	
		Non	2				
		Ne sait pas	9				
**(2.14)**	L’agent de santé a-t-il demandé si l’enfant vomit tout ce qu’il prend ?	1	2			|__|	
	**Si (2,14)=1, L’enfant vomit tout ce qu’il prend?**	Oui	1			|__|	
		Non	2				
		Ne sait pas	9				
**(2.15)**	L’agent de santé a-t-il demandé si l’enfant est léthargique ou s’il y a un changement dans son niveau de conscience ?	1	2			|__|	
	**Si (2,15)=1, L’enfant est léthargique ou a eu un changement dans son niveau de conscience?**	Oui	1			|__|	
		Non	2				
		Ne sait pas	9				
**(2.16)**	L’agent de santé a-t-il demandé si l’enfant avait convulsé ?	1	2			|__|	
	**Si (2,16)=1, L’enfant a convulse’?**	Oui	1			|__|	
		Non	2				
		Ne sait pas	9				
**(2.17)**	L’agent de santé a-t-il demandé si l’enfant a la diarrhée ?	1	2	►	**(2.21)**	|__|	
**(2.18)**	Notez ce que le patient a répondu à ce sujet : l’enfant a-t-il la diarrhée ?	1	2	►	**(2.21)**	|__|	
**(2.19)**	L’agent de santé a-t-il demandé depuis quand le patient a la diarrhée ?	1	2			|__|	
	**Si (2,19)=1, Depuis quand le patient a la diarrhée ?**	a. jours (99 ne sait pas)				|__|__|	
		b. Mois (99 ne sait pas)				|__|__|	
**(2.20)**	L’agent de santé a-t-il demandé s’il y a du sang dans les selles ?	1	2			|__|	
	**Si (2,20)=1, Est-ce qu’il y a du sang dans les selles ?**	Oui	1			|__|	
		Non	2				
		Ne sait pas	9				
**(2.21)**	L’agent de santé a-t-il vérifié les plis cutanés ?	1	2			|__|	
	**Si (2,21)=1,OBSERVEZ: Le pli cutané s’efface normalment, un peu lentement, ou tres lentement?**	Normalement	1			|__|	
		Un peu lentement	2				
		Très lentement	3				
		Ne sait pas	9				
**(2.22)**	Quel est le diagnostic syndromique probable mentionné par l’agent de santé pour la diarrhée de l’enfant ?	a. Aucun diagnostic mentionné				|__|	
		b. Déshydratation sévère				|__|	
		c. Déshydratation modérée				|__|	
		d. Pas de déshydratation				|__|	
	NOTEZ 1 POUR OUI ET 2 POUR NON	e. Dysenterie probable				|__|	
		f. Autre à préciser				|__|	
**(2.23)**	L’agent de santé a-t-il demandé si l’enfant a la toux ou des difficultés respiratoires ?	Oui	1			|__|	
		Non	2	►	**(2.26)**		
**(2.24)**	Le patient a-t-il la toux ou des difficultés respiratoires ?	Oui	1			|__|	
		Non	2	►	**(2.26)**		
**(2.25)**	L’agent de santé a-t-il demandé depuis quand le patient a la toux ou des difficultés respiratoires ?	1	2			|__|	
	**Si (2,25)=1, Depuis quand le patient a la toux ou des difficultés respiratoires ?**	a. jours (99 ne sait pas)				|__|__|	
		b. Mois (99 ne sait pas)				|__|__|	
**(2.26)**	L’agent de santé a-t-il demandé si l’enfant a le stridor ou un sifflement durant la respiration?	1	2			|__|	
	**Si (2,26)=1, L’enfant a le stridor ou un sifflement durant la respiration?**	Oui	1			|__|	
		Non	2				
		Ne sait pas	9				
**(2.27)**	L’agent de santé a-t-il vérifié la fréquence respiratoire ?	1	2			|__|	
	**Si (2,27)=1, OBSERVEZ: Est-ce que la frequence respiratoire est normale ou rapide?**	Normale	1			|__|	
		Rapide	2				
		Ne sait pas	9				
**(2.28)**	L’agent de santé a-t-il déshabillé l’enfant ?	1	2			|__|	
	**Si (2,28)=1, L’enfant a un tirage sous-costal (mouvement vers l’intérieur de la structure osseuse de la paroi thoracique à l’inspiration)?**	Oui	1			|__|	
		Non	2				
		Ne sait pas	9				
**(2.29)**	L’agent de santé a-t-il ausculté l’enfant ?	1	2			|__|	
**(2.30)**	Quels sont les diagnostics posés par l’agent de santé pour la toux/difficulté respiratoire de l’enfant ?	a. Aucun diagnostic mentionné	, 1►(2.31)			|__|	
		b. Pneumonie grave				|__|	
	NOTEZ 1 POUR OUI ET 2 POUR NON	c. Bronchiolite				|__|	
		d. Pneumonie simple				|__|	
						|__|	
		f. Autre, préciser				|__|	
**(2.31)**	L’agent de santé a-t-il demandé si l’enfant a eu de la fièvre pendant les 24 dernières heures ?	Oui	1			|__|	
		Non	2	►	**(2.35)**		
**(2.32)**	Le patient a-t-il eu la fièvre au cours des 24 dernières heures ?	Oui	1			|__|	
		Non	2	►	**(2.35)**		
**(2.33)**	L’agent de santé a-t-il vérifié la température si ce n’était pas encore fait par un agent avant ?	1	2			|__|	
	**Si (2,33)=1, Température de l’enfant?**						
		Température en degré celsius (ne sait pas: 99.99)				**|__|__|.|__|**	
**(2.34)**	L’agent de santé a-t-il demandé depuis quand le patient a de la fièvre ?	1	2			|__|	
	**Si (2,34)=1, Depuis combien de temps l’enfant a fievre ? (99 ne sait pas)**	a. jours (99 ne sait pas)				|__|__|	
		b. Mois (99 ne sait pas)				|__|__|	
**(2.35)**	L’agent de santé a-t-il demandé si l’enfant a eu la rougeole avant ?	1	2			|__|	
	**Si (2,35)=1, L’enfant a eu la rougeole avant?**	Oui	1			|__|	
		Non	2				
		Ne sait pas	9				
**(2.36)**	L’agent de santé a-t-il vérifié l’état de la fontanelle (pour les patients de moins de 8 mois, pour les autres Notez 9) ?	1	2		9	|__|	
	**Si (2,36)=1, La fontanelle de l’enfant est bombée?**	Oui	1			|__|	
		Non	2				
		Ne sait pas	9				
**(2.37)**	L’agent de santé a-t-il examiné les yeux du patient ?	1	2			|__|	
	**Si (2,37)=1, Les yeux de l’enfant sont enfoncés?**	Oui	1			|__|	
		Non	2				
		Ne sait pas	9				
**(2.38)**	L’agent de santé a-t-il regardé si le nez coulait ?	1	2			|__|	
	**Si (2,38)=1, Le nez de l’enfant coulait?**	Oui	1			|__|	
		Non	2				
		Ne sait pas	9				
**(2.39)**	L’agent de santé a-t-il regardé si l’enfant a une éruption cutanée ?	1	2			|__|	
	**Si (2,39)=1, L’enfant a une éruption cutanée?**	Oui	1			|__|	
		Non	2				
		Ne sait pas	9				
**(2.40)**	L’agent de santé a-t-il regardé la gorge de l’enfant?	1	2			|__|	
	**Si (2,40)=1, L’enfant a une gorge enflammée?**	Oui	1			|__|	
		Non	2				
		Ne sait pas	9				
		a. Aucun diagnostic mentionné				|__|	
**(2.41)**	Quel est le(s) diagnostic(s) posé(s) par l’agent de santé pour la fièvre de l’enfant ?	b. Otite				|__|	
		c. Paludisme				|__|	
		d. Grippe				|__|	
		e. Rhinite				|__|	
	NOTEZ 1 POUR OUI ET 2 POUR NON	f. Rougeole compliquée grave				|__|	
		g. Rougeole				|__|	
		h. Amigdalite/Angine				|__|	
		i. Autre à préciser				|__|	
**(2.42)**	L’agent de santé a-t-il regardé dans les oreilles ?	Oui	Non			|__|	
		1	2			|__|	
	**Si (2,42)=1, L’enfant a une infection de l’oreille?**	Oui	1			|__|	
		Non	2				
		Ne sait pas	9				
**(2.43)**	L’agent de santé a-t-il regardé derrière les oreilles ?	1	2			|__|	
	**Si (2,43)=1, L’enfant a un gonflement douloureux derrière l’une des oreilles?**	Oui	1			|__|	
		Non	2				
		Ne sait pas	9				
**(2.44)**	L’agent de santé a-t-il demandé si l’enfant a une douleur ou un écoulement au niveau de l’oreille ?	1	2			|__|	
	**Si (2,44)=1, L’enfant a mal à l’oreille?**	Oui	1			|__|	
		Non	2				
		Ne sait pas	9				
	**Si (2,44)=1, L’enfant a un écoulement (pus ou autre) dans l’oreille?**	Oui	1			|__|	
		Non	2				
		Ne sait pas	9				
**(2.45)**	L’agent de santé a-t-il vérifié les yeux de l’enfant ou la paume des mains, ou la plante des pieds (a la recherche de l’anémie) ?	1	2			|__|	
	**Si (2,45)=1, L’enfant souffre d’anémie?**	Oui	1			|__|	
		Non	2				
		Ne sait pas	9				
**(2.46)**	L’agent de santé a-t-il regardé les deux pieds ou les deux chevilles (a la recherche de l’oedème) ?	1	2			|__|	
	**Si (2,46)=1, L’enfant a des œdèmes?**	Oui	1			|__|	
		Non	2				
		Ne sait pas	9				
**(2.47)**	L’agent de santé a-t-il dit que l’enfant souffre d’une malnutrition moderée ou de malnutrition sevère?	1	2			|__|	
	**Si (2,47)=1, L’enfant souffre de malnutrition?**	Oui, malnutrition modere	0			|__|	
		Oui, malnutrition sevère	1				
		Ne sait pas	9				
**(2.48)**	L’agent de santé a-t-il fait un test de paludisme?	1	2			|__|	
	**Si (2,48)=1, Le test est positif – l’enfant a paludisme**	Oui	1			|__|	
		Non	2				
		Ne sait pas	9				
**(2.49)**	La porte était-elle fermée ou le rideau était-il tiré pour garantir la confidentialité du patient ?	1	2			|__|	
***Section 3 : Counseling***							
	NOTER « 1 » POUR OUI ; « 2 » POUR NON POUR CHACUNE DES OPTIONS SUIVANTES	**Oui**	**Non**				
**(3.01)**	L’agent de santé a-t-il donné le nom de la maladie à la mère / l’accompagnant ?	1	2			|__|	
**(3.02)**	L’agent de santé a-t-il expliqué la maladie, ses causes et/ou son évolution à la mere ou son accompagnant ?	1	2			|__|	
**(3.03)**	L’agent de santé a-t-il dit ce que devrait faire la mère / l’accompagnant à la maison pour l’enfant ?	Oui	1			|__|	
		Non	2	►	**(3.05)**		
**(3.04)**	**L’agent de santé recommande t-il :**	**Oui**	**Non**				
**a**	de donner plus de liquides	1	2			|__|	
**b**	de continuer ou d’augmenter l’alimentation et/ou l’allaitement (pour les enfants de **moins de 6 mois allaitement exclusif**)	1	2			|__|	
**c**	de donner les bains froids/enveloppement humide pour la fièvre	1	2			|__|	
**d**	éviter qu’il prenne froid	1	2			|__|	
**e**	d’éviter les médicaments autres que ceux prescrits aujourd’hui	1	2			|__|	
**f**	Autre à préciser	1	2			|__|	
**(3.05)**	L’agent de santé a-t-il donné à la mère / l’accompagnant une prescription ou un médicament à administrer aujoud’hui à la maison ?	Oui	1			|__|	
		Non	2	►	**(3.07)**		
**(3.06)**	**L’agent de santé :**	**Oui**	**Non**				
**a**	Dit-il le nom du médicament à la mère /l’accompagnant ?	1	2			|__|	
**b**	Explique t-il la posologie ?	1	2			|__|	
**c**	Dit-il quels peuvent être les effets secondaires et ce qu’il faut faire ?	1	2			|__|	
**(3.07)**	Indique t-il à la mère /l’accompagnant les signes ou symptômes qui pourraient montrer l’agravation de la maladie?	Oui	1			|__|	
		Non	2	►	**(3.09)**		
**(3.08)**	**L’agent de santé mentionne-t-il la conduite à tenir si:**	**Oui**	**Non**				
**a**	la fièvre ne disparaît pas après un certain temps	1	2			|__|	
**b**	la fièvre augmente	1	2			|__|	
**c**	l’enfant est incapable de boire ou ne boit pas bien	1	2			|__|	
**d**	il y a un changement au niveau de la conscience	1	2			|__|	
**e**	la diarrhée persiste	1	2			|__|	
**f**	du sang apparaît dans les selles	1	2			|__|	
**g**	l’enfant développe une respiration rapide ou difficile	1	2			|__|	
**h**	l’enfant devient plus malade pour n’importe quelle raison	1	2			|__|	
**i**	de nouveaux symptômes se développent	1	2			|__|	
**j**	Autre à préciser	1	2			|__|	
**(3.09)**	**L’agent de santé**	**Oui**	**Non**				
**a**	mentionne-t-il à la mère / l’accompagnant une date pour un contrôle programmé (visite de contrôle) ?	1	2			|__|	
**b**	dit-il à la mère /l’accompagnant d’aller dans une autre formation sanitaire (avec une lettre de référence) ?	1	2			|__|	
**c**	explique-t-il la raison de la référence ?	1	2			|__|	
**d**	demande-t-il si la mère / l’accompagnant a des questions à la fin de la visite?	1	2			|__|	
**e**	vérifie-t-il le carnet de vaccination de l’enfant ?	1	2			|__|	
**f**	recommande-t-il d’aller faire une vaccination ?	1	2			|__|	
**g**	dit-il à la mère /l’accompagnant d’amener l’enfant pour des examens de laboratoire ?	1	2			|__|	
**h**	dit-il à la mère /l’accompagnant si l’enfant doit rester dans la formation sanitaire pour traitement ou observation?	1	2			|__|	
**(3.10)**	**L’agent de santé :**	**Oui**	**Non**				
**a**	remplit-il une fiche de consultation?	1	2			|__|	
**b**	fait-il un enregistrement de l’enfant dans le registre ?	1	2			|__|	
**(3.11)**	se lave t-il les mains avec du savon et de l’eau du robinet ou seau avec robinet après avoir examiner le patient ?	Oui	1			|__|	
		Non	2				
**(3.13)**	Heure de fin de la consultation ex. 7 h est 0700, 8 h 30 est 0830 et 19 h est 1900					|__|__|	
**(3.14)**	**TOTAL** temps passé (MINUTES) en consultation					|__|__|	
**(3.15)**	Langue de la consultation	Français	1			|__||__|	
		Lingala	2				
		Kikongo	3				
		Swahili	4				
		Autre, à préciser	97				

## Abbreviations

DRC: Democratic Republic of Congo
IMCI: Integrated Management of Childhood Illness
PBF: Performance-based financing

## Footnotes

1 UNFPA State of the World’s Midwifery 2014 report.
